# The Impact of Negative Emotions on the Treatment Outcome of Percutaneous Balloon Compression for Idiopathic Trigeminal Neuralgia Patients: A Longitudinal Study

**DOI:** 10.1155/da/5535907

**Published:** 2025-06-03

**Authors:** Chengrong Jiang, Yulong Chong, Chenjun Jiang, Weibang Liang, Chunran Zhu

**Affiliations:** ^1^Department of Neurosurgery, Nanjing Drum Tower Hospital Clinical College of Traditional Chinese and Western Medicine, Nanjing University of Chinese Medicine, Nanjing 210009, Jiangsu, China; ^2^Department of Diagnostic Clinical Oncology, LKS Faculty of Medicine, The University of Hong Kong, 21 Sassoon Road, Pokfulam, Hong Kong SAR, China; ^3^Affiliated Hospital of Integrated Traditional Chinese and Western Medicine, Nanjing University of Chinese Medicine, Nanjing 210028, Jiangsu, China

**Keywords:** depression, mental health, negative emotions, PBC surgery, QoL, trigeminal neuralgia

## Abstract

**Background:** Trigeminal neuralgia (TN) is a common yet severe facial pain condition. Percutaneous balloon compression (PBC) is a widely promoted surgical treatment method for TN due to its simplicity and effectiveness. However, patients who undergo PBC present with varying degrees of depression. This study aims to investigate the depressive factors affecting TN patients following PBC.

**Methods:** The Penn-FPS Scale, Hamilton Anxiety Scale (HAMA), Hamilton Depression Scale (HAMD), BNI Facial Numbness Scale, Pittsburgh Sleep Quality Index (PSQI), and Life Satisfaction Index-B (LSI-B) scale were used to evaluate depressive symptoms in TN patients before and after PBC, as well as at various time points during a 1-year follow-up. Factors impacting postoperative quality of life (QoL) were identified and evaluated.

**Findings:** Depressive symptoms improved significantly following PBC treatment; however, the presence of these symptoms could lead to delays in TN recovery. Patients with higher preoperative pain score and longer TN course showed better tolerance of numbness following PB surgery. Meanwhile, the degree of numbness was positively related to the duration of balloon compression, while previous treatments for TN were associated with increased risk of depression and anxiety.

**Conclusions:** PBC is an effective surgery for TN patients. Depressive disorders impact prognosis and should be carefully managed as part of a comprehensive treatment plan to improve the QoL following PBC.

**Summary**

• This study is a single-center prospective study with a 12-month follow-up of idiopathic trigeminal neuralgia (TN) cases treated with percutaneous balloon compression (PBC). The study aims to investigate the clinical efficacy of PBC and to provide a reference for treating TN patients with pain comorbidities.
• Overall, PBC demonstrates good clinical efficacy for the treatment of TN. Patients experienced pain relief after the surgery and their quality of life (QoL) generally improved over time.
• It was found that the degree of preoperative pain comorbidities, including anxiety and depression, led to greater challenges in postoperative recovery.
• High severity of preoperative pain and long TN course were associated with better acceptance of numbness after PBC surgery.
• The duration of intraoperative balloon compression was related to a higher degree of numbness after surgery.
• Patients who had undergone previous treatments for TN, such as radiofrequency or microvascular decompression (MVD) surgery, presented with a lower QoL after PBC treatment.
• PBC has good clinical efficacy for the treatment of TN. Most patients experienced pain relief after the surgery and their QoL improved overall. For patients with preoperative depression or previous TN treatments, explaining postoperative outcomes such as facial numbness can help them understand and better adapt to these conditions. We also recommend that physicians control the balloon compression time for better postoperative recovery.

## 1. Background

Trigeminal neuralgia (TN) is a common chronic pain condition characterized by sudden, severe, and recurrent facial pain affecting the distribution of the trigeminal nerve [1]. Current research on TN primarily focuses on pain management modalities; however, in recent years, attention has shifted toward addressing mental disorders in TN patients. Previous studies have linked chronic pain to the occurrence of depressive symptoms, while the presence of anxiety and depression can exacerbate pain perception [2–5]. The incidence of pain comorbidities among TN patients, especially depressive disorders, has been found to be three times higher than that in the general population worldwide [6]. In European TN patients, the incidence of depression can be as high as 33% [7], while in Asian TN patients, this figure is reported to be 37%, significantly higher than in patients with other chronic diseases [8, 9]. A large sample cohort study based on pain and depression questionnaires has also shown that over half of TN patients had anxiety and over a third presented with mild-to-severe depression [10]. These pain-related comorbidities are also prevalent among TN patients treated at our institution, significantly impacting their quality of life (QoL). Preoperative depression and anxiety are common in patients prior to surgery and have been shown to increase pain perception by lowering the pain threshold, which is associated with adverse surgical outcomes [11]. On the other hand, postoperative depressive symptoms not only negatively affect surgical results [12], but are also risk factors for long-term incomplete recovery due to chronic inflammatory responses that impede postoperative trauma healing cascades [13, 14]. In some cases, depression and anxiety can persist in patients for a year after surgery, increasing the risk of life dissatisfaction and further hospitalizations [12].

Percutaneous balloon compression (PBC) is a promising treatment especially for patients with poor physical conditions or recurrent TN after microvascular decompression (MVD) [15]. In 1978, Mullan and Lichtor [16] pioneered the PBC procedure based on the theoretical foundation laid by Crue et al. [17]. PBC has since become the most common minimally invasive treatment for TN worldwide, owing to its high efficacy and simple operation [15, 18–20]. With the global increase in TN patients undergoing PBC surgery, its effectiveness and safety have been confirmed by numerous clinical and research studies from medical institutions [21–25]. PBC was first introduced in China in 2000, with Ma et al. [26] reporting its use in 2002. In recent years, PBC has gained widespread recognition in China and has become one of the primary surgical treatments for TN [27].

While studies have evaluated QoL among TN patients who have received MVD, radiofrequency therapy, and other treatments [20, 21, 28, 29], assessments of QoL among TN patients receiving PBC remain scarce. Research indicates that pain relief from TN often correlates with improvements in pain-related comorbidities [30]; however, these comorbidities may persist after treatment and are associated with lower QoL, leading to challenges in postoperative recovery [31]. In addition to direct measurements using the Hamilton Anxiety Scale (HAMA) and Hamilton Depression Scale (HAMD) [32], depression and anxiety are often linked to disturbed sleep quality which can be assessed using the self-reported Pittsburgh Sleep Quality Index (PSQI) [33]. Facial numbness, another common factor affecting QoL in TN patients, can be evaluated using the BNI Facial Numbness Scale [34]. Meanwhile, Life Satisfaction Index-B (LSI-B) quantifies overall QoL [35], while the Penn-FPS scale quantifies the impact of TN pain [36].

Since the implementation of PBC at our institution, we have observed varying degrees of improvement in TN patients following treatment, influenced by preoperative and postoperative pain levels, perceptions of postoperative numbness, durations of TN, and treatment histories. Although the impact of anxiety and depression on PBC patients' QoL was evident, it has been challenging for physicians to take appropriate actions. Furthermore, there is a lack of knowledge regarding the risk factors for anxiety and depressive symptoms, how these factors affect postoperative emotions and QoL, and their association with pain perception. This research aims to analyze the effects of individual factors on depressive symptoms in TN patients treated with PBC, providing guidelines and references to improve surgical outcomes.

## 2. Methods

### 2.1. Study Subjects

This study was approved by the Ethical Committee of Nanjing Drum Tower Hospital and the ethics approval number is 2024-JS-01. All research protocols were carried out under approved institutional guidelines and regulations. Informed consent was obtained from TN patients who were hospitalized in the Department of Neurosurgery at Nanjing Drum Tower Hospital from April 2021 to August 2022 and underwent PBC surgery. The cases were included in this study based on the inclusion criteria listed in the next section.

The level of preoperative TN pain, level of anxiety, depression, sleep quality, and life satisfaction were measured using the Penn-FPS scale, HAMA, HAMD, PSQI, and LSI-B, respectively, during the preoperative period before PBC surgery [32–36]. Subjects were grouped according to these assessment results.

**Figure 1:**
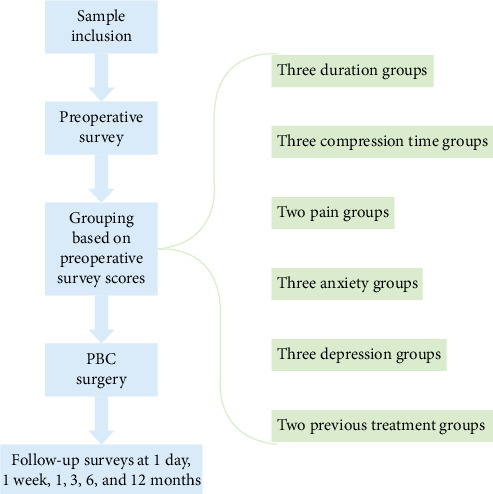
Study flow diagram. Following sample inclusion, the patients were grouped based on the preoperative survey results. The PBC surgery was conducted and the follow-up results were compared between the groups.

All PB surgeries were performed by the same chief physician, who was blinded to these results. Preoperative assessments were conducted by one group of physicians, while postoperative assessments, follow-ups, and data analysis were conducted by a separate group. The physicians responsible for scoring underwent standardized training and were blinded to the patients' diagnosis and treatments. Postoperative levels of pain, anxiety, depression, facial numbness, sleep quality, and life satisfaction were measured with the Penn-FPS scale, HAMA, HAMD, BNI Facial Numbness Scale, PSQI, and LSI-B at 1 day, 1 week, 1, 3, 6, and 12 months after PBC surgery. The study procedures are summarized in Figure 1.

### 2.2. Inclusion Criteria

1. Patients diagnosed with idiopathic TN according to the criteria described in the International Classification of Headache Disorders (ICHD) third edition [1], shown in Tables 1 and 2.
2. Patients had no abnormalities in cardiac or pulmonary function, no surgical contraindications, and were prescribed PBC treatment.
3. Patients had no cognitive impairments, were willing to participate and cooperate in the clinical study, and to sign the consent form.
4. Patients could ensure unimpeded communication during follow-up.

### 2.3. Exclusion Criteria

1. Patients with secondary TN, with a history of herpes virus infection, or concurrent multiple sclerosis or intracranial tumors or other secondary TN causative factors.
2. Patients who were diagnosed with mental disorders before TN occurrence.
3. Patients presented unsuccessful PBC puncture or balloon rupture during surgery; poor compliance, serious mental disorders, or unable to provide normal clinical results.
4. Intellectual and communication disorders, unable to engage in language communication.

### 2.4. Surgical Method

**Figure 2:**
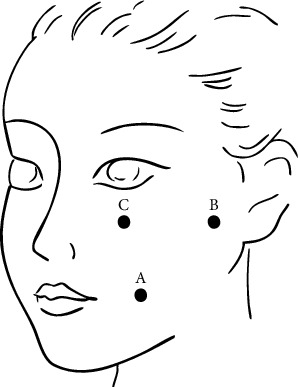
Puncture point reference diagram. Point A: 2.5 cm lateral to the corner of the mouth; Point B: located below the zygomatic arch, 3.0 cm anterior to the external auditory canal; Point C: intersection of the line from point A to the pupil and the lower edge of the orbit.

1. Before anesthesia, the TN-affected side and range were checked. Equipment and other preoperative preparations were carefully checked.
2. Patients went through general anesthesia and arterial pressure monitoring, as intraoperative trigeminal–cardiovascular reflexes may induce dramatic fluctuations in blood pressure and heart rate. This ensures effective monitoring of intraoperative changes and surgical safety.
3. After successful anesthesia, patients were placed in the supine position for PBC surgery. A triangular bandage was used to create a circular pillow at the occipital region to prevent head displacement during the surgery. The puncturing location was determined such that it was 3 cm anterior to the external ear, below the pupil, and lateral to the corner of the mouth (Figure 2). Eye patches were used to protect the cornea from irritation by disinfectant during surgery.
4. Before starting the surgery, a lateral skull X-ray or fluoroscopy was taken to ensure that the beam was perpendicular to the skull and that the bony structures such as the bilateral zygomatic arches are symmetrically overlapped. The head was slightly tilted backward and the conventional Hartel's puncture approach was used.
5. A scalpel blade was used to make a 1–2 mm incision at point A and a 5F balloon puncture sheath needle was used for the puncture. After puncturing the skin, the mandible was palpated and the needle was inserted along the inner edge of the mandible, taking care to avoid contamination by entering the oral cavity. Generally, a depth of 6–7 cm was reached at the middle cranial fossa and X-ray localization was performed again to clarify the direction and depth of the puncture needle. The puncture needle was adjusted according to the imaging results until the needle entered the foramen ovale. After puncturing the Meckel's cave (MC) with the needle core, the core was drawn slowly, taking care to fix the puncture needle to prevent displacement. Then a disposable neurosurgical balloon catheter was inserted with the catheter tip generally extending about 1 cm beyond the puncture needle sheath. Contrast agent was slowly injected and the catheter depth and morphology were determined through fluoroscopy. The insertion during surgery was controlled strictly to avoid the catheter tip exceeding 5 mm beyond the petrous slope line from the lateral view to avoid brain stem injury or adverse consequences.
6. The depth and balloon size were adjusted according to the actual situation until a pear-shaped balloon was formed with a pointed end facing the posterior cranial fossa. The balloon pressure was maintained between 150 and 170 kPa. During the process, fluctuations in the patient's heart rate and blood pressure were closely monitored and the anesthesiologist cooperated as much as possible to ensure the stability of blood pressure and heart rate during surgery. After reaching the estimated compression time, the balloon was withdrawn, marking the completion of the surgery.

### 2.5. Efficacy Evaluation and Follow-up

A questionnaire was developed based on relevant literature [32–36]. Trained physicians surveyed the patients and collected the filled-out questionnaires. Follow-ups were conducted via phone calls or visits. The survey content included: Penn-FPS scale, HAMA, HAMD, BNI Facial Numbness Scale, PSQI LSI-B, and other related scales. Among them, PSQI included seven items: sleep duration, sleep efficiency, subjective sleep quality, sleep disturbances, time to fall asleep, hypnotic medication use, and daytime energy condition, with a total score of 21. A score of <7 indicates good sleep quality, while a score of >7 indicates sleep disturbances. The higher the score, the worse the sleep quality. Meanwhile, HAMD scores of <8 indicate no depression, 8–20 indicate possible depression, >20 indicate depression, and >35 indicate severe depression. The survey was conducted: before surgery, 1 day after surgery, 1 week after surgery, 3 months after surgery, 6 months after surgery, and 12 months after surgery, and the survey data were collected. All scores were scaled for evaluation of postoperative QoL.

### 2.6. Statistical Methods

Categorical variables were expressed as frequency (%) and continuous variables were expressed as mean ± standard deviation (SD). Independent sample *t*-tests were used for comparisons between two groups and analysis of variance (ANOVA) was used for comparisons between three groups. The Fisher's least significant difference (LSD) method Dunnett T3 was used for comparisons of pairwise variables presenting differences between groups during the follow-up. The results were represented using lowercase letter subscript endings by default. All statistical tests were two-sided, with *p*-values less than 0.05 considered statistically significant. Data analysis was performed using SPSS 26.0 version (IBM, USA).

### 2.7. Role of the Funding Source

There was no funding source for this study. All authors had access to the data and all authors are responsible for the decision to submit the manuscript for publication.

## 3. Results

A total of 164 samples were included in the study, with an average age of 67.74 years. Among them, 65 were males (39.6%) and 99 were females (60.4%). The number of patients with right-sided TN was 99 (60.4%), compared to 64 (39.0%) with left-sided TN. Only one case had bilateral TN. A total of 79 (48.2%) patients had hypertension and 140 (85.3%) had diabetes. Two patients relapsed during follow-up and two patients with insignificant postoperative pain relief were excluded from the sample. The overall effectiveness rate of PBC was as high as 97.6%. After surgery, 63 (38.7%) patients experienced herpes labialis that recovered within a week. Hearing loss occurred in two (1.2%) patients. Eighty-nine (54.6%) patients experienced various levels of decrease in bite force and the conditions later improved. An overview of the sample population can be found in Table 3, while the quartiles and ranges of disease duration and intraoperative balloon compression time are detailed in Table 4.

**Figure 3:**
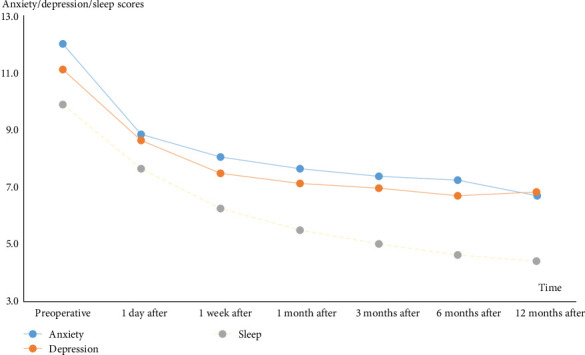
Preoperative and postoperative anxiety, depression, and sleep scores at various time points. The levels of anxiety, depression, and sleep disturbance decreased after PBC surgery and throughout the follow-up period, indicating overall improvements in these factors.

**Figure 4:**
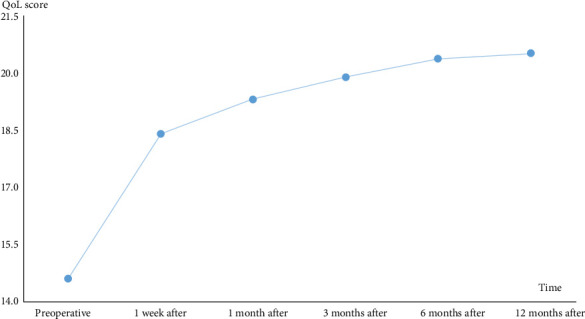
Preoperative and postoperative QoL scores at various time points. The average QoL score increased following PBC surgery and continued to rise throughout the 12-month follow-up period.

The proportion of TN patients who experienced negative emotional status, such as anxiety and depression, was much higher than that in the general population, impacting the health-related QoL. After undergoing PBC surgery, patients experienced pain relief and both preoperative and postoperative anxiety, depression, and sleep scores improved over time, leading to an overall enhancement in QoL (Figures 3 and 4).

The sample population was divided into three duration groups based on the duration of TN: duration ≤ 1 year (*N* = 21), 1 < duration ≤ 5 years (*N* = 73), and >5 years (*N* = 70). The degrees of anxiety, depression, sleep quality, numbness, numbness acceptance, and QoL at 1 day, 1 week, 1, 3, 6, and 12 months postsurgery were analyzed (Tables 5–10). The LSD method (Dunnett T3) was employed for pairwise comparisons and the results were represented using lowercase letter subscript endings. Items with the same letter have no statistically significant difference between them (e.g., “a” and “a”), while different letters signify a statistically significant difference (e.g., “a” and “b”). Significant differences were noted in numbness acceptance among the three groups on postoperative day 1 (*p*=0.042) and in the level of numbness among the three groups at 3 months (*p*=0.007) and 12 months after surgery (*p*=0.048).

The sample population was then divided into three compression time groups based on their intraoperative balloon compression time: compression time < 90 s (*N* = 42), 90 < compression time ≤ 120 s (*N* = 64), and compression time > 120 s (*N =* 58). The degrees of anxiety, depression, sleep quality, numbness, numbness acceptance, and QoL were analyzed during follow-up at 1 day, 1 week, 1, 3, 6, and 12 months postsurgery (Tables 11–16). The level of anxiety (*p*=0.014, *F* = 4.398), numbness (*p* < 0.001, *F* = 15.213), and numbness acceptance (*p*=0.024, *F* = 3.829) varied significantly on postoperative day 1 and at 6 months (*p*=0.038, *p* < 0.001, and *p*=0.008, respectively) after PBC. QoL was also significantly different between the compression time groups from 1 week (*p*=0.001, *F* = 7.531) to 6 months (*p*=0.008, *F* = 4.992) after surgery, while at 12 months after surgery, only sleep quality varied among groups (*p*=0.025, *F* = 3.857).

The sample population was divided into two preoperative pain groups (pain ≤ 110 points, *N* = 23 and pain > 110 points, *N* = 141) according to preoperative pain scores. The levels of postoperative anxiety, depression, sleep quality, numbness, numbness acceptance, and QoL at 1 day, 1 week, 1, 3, 6, and 12 months after PBC surgery were compared between the two groups (Tables 17–22). Although the level of numbness showed no difference between the two groups, the group with pain > 110 points showed better tolerance of numbness at 3, 6, and 12 months after PBC surgery (*p*=0.041, *p*=0.005, and *p*=0.023, respectively), with notably lower anxiety scores at 12 months (*p*=0.043, *F* = 4.195).

The sample population was divided into three preoperative anxiety groups based on their preoperative anxiety scores: anxiety score ≤ 8 (*N* = 72), 8 < anxiety score ≤ 20 (*N* = 73), and anxiety score > 20 (*N* = 19). The levels of anxiety, depression, sleep quality, numbness, numbness acceptance, and QoL at 1 day, 1 week, 1, 3, 6s, and 12 months after surgery were compared (Tables 23–28). Except numbness which showed no significant difference between the three groups, other indicators including anxiety (*p* < 0.001), depression (*p* < 0.001), sleep (*p* < 0.001), and numbness acceptance (*p* < 0.001) differed among the three groups on postoperative day 1. At 1 week, 3, 6, and 12 months, all differences remained except in numbness scores where *p* > 0.05.

The sample population was divided into three preoperative depression groups according to the preoperative depression scores: depression score ≤ 8 (*N* = 80), 8 < depression score ≤ 20 (*N* = 66), and depression score > 20 (*N* = 18). The levels of anxiety, depression, sleep quality, numbness, numbness acceptance, and QoL at 1 day, 1 week, 1, 3, 6, and 12 months after surgery were compared (Tables 29–34). It was found that except for the level of numbness which had *p*  > 0.05, all other indicators including the level of anxiety, depression, sleep, numbness acceptance, and QoL were significantly different (*p*  < 0.001) among the three preoperative depression groups, and this trend persisted throughout the follow-up from day 1 to 12 month after PBC.

According to whether the patient had previous treatment failures (*N* = 106) such as MVD and radiofrequency treatment, et cetera, or not (*N* = 58) in terms of TN treatment, the population was divided into two preoperative treatment groups to compare for the degrees of anxiety, depression, sleep quality, numbness, numbness acceptance, and QoL at 1 day, 1 week, 1, 3, 6, and 12 months after surgery (Tables 35–40). During the follow-up, it was found that for the group having previous treatments from 1 day to 6 months after PBC surgery, the level of anxiety was significantly higher than in the group having no previous treatments (*p* < 0.05) and the level of depression was also significantly higher at 1 week (*p*=0.003, *F* = 9.167), 1 month (*p*=0.012, *F* = 6.526), 3 months (*p*=0.016, *F* = 5.944), and 6 months (*p*=0.029, *F* = 4.874) after PBC. The postoperative QoL in the group without previous treatments was also found to be higher in the group without previous treatment than that in the group received previous treatments at 1 week (*p*=0.015, *F* = 5.997), 1 month (*p*=0.026, *F* = 5.015), and 3 months (*p*=0.045, *F* = 4.067) after PBC.

## 4. Discussion

A total of 12 months of follow-up was conducted with 164 TN patients to evaluate the depression-related factors before and after PBC treatment. The study population primarily consisted of middle-aged and elderly individuals, which aligns with the demographic incidence age of TN [36–39]. The slightly higher female ratio was consistent with the higher female-to-male risk of TN [40, 41], while the higher proportion of right-sided TN is consistent with TN epidemiology [42, 43]. A large proportion of the sample population also had hypertension and diabetes. This high prevalence of chronic disorders may be attributed to the long-term effects of chronic disease-induced vascular changes and compression at the trigeminal nerve root, which predispose individuals to the development of TN [44]. Research indicated that the most common vessels responsible for TN predisposing neurovascular compression are the superior cerebellar artery (SCA), followed by the anterior and posterior inferior cerebellar artery (AICA and PICA), the vertebral artery, and a small portion of cases triggered by venous compression [45, 46].

Overall improvements were observed in pain levels, anxiety, depression, sleep quality, and QoL scores after PBC surgery. The long-term benefits suggest that effective pain relief can lead to a reduction in depressive symptoms over time, confirming that PBC is an effective treatment for TN [18]. However, patients still exhibited significantly higher level of depression and anxiety than the general population, corroborating findings from previous studies [3, 10]. The result shows that preoperative anxiety is a key factor delaying prognostic recovery. Given that TN could act as a trigger of depression [6, 7], it is unlikely that depressive symptoms will diminish immediately following PBC. This study found that increased depression and anxiety were associated with a decreased acceptance of postoperative numbness. Thus, higher anxiety and depression scores before and after surgery correlated with challenges in accepting postoperative numbness, resulting in less improvement in QoL. Previous researches have reported that depressive emotions can impact pain perception by increasing pain sensitivity [30, 31], while heightened pain perception can intensify negative emotions [7, 47]. Additionally, studies suggest that depression and pain share neurobiological pathways, including pro-inflammatory cytokines and neurotransmitters [2, 10, 30]. Thus, treating both conditions simultaneously could be beneficial. We recommend a comprehensive treatment approach for TN patients suffering from depressive disorders.

Another important finding was the positive correlation between the duration of TN from onset to PBC and the level of numbness acceptance after surgery. We suspect that those who have suffered from facial pain for a longer period may be better able to accept numbness as a substitute for pain. Thus, patients with a longer duration of TN showed relatively better acceptance of numbness. This result aligns with findings from the pain groups, where we observed that higher preoperative pain scores were associated with greater acceptance of numbness following PBC. Similarly, we infer that individuals who experienced more intense pain before PBC treatment are more likely to accept numbness as a substitute for pain. Therefore, while numbness can be bothersome after PBC, it seems to be better accepted by those who have endured longer and more severe TN progressions.

Additionally, results from the compression time groups indicated a significant relationship between longer intraoperative balloon compression time and the degree of post-PBC numbness. To minimize numbness, Lichtor and Mullan [48] recommended a 1-min compression inflating the balloon until a pear shape appears, rather than prolonged compression. Although there is no firm consensus on the optimal balloon compression time, most studies suggest that 1–2 min is sufficient for TN and extended balloon compression is associated with a higher risk of numbness and postoperative facial discomfort [13, 14, 49]. Our finding highlighted the importance of controlling PBC compression time, which should be considered as part of the individualized treatment during surgical planning, and the physician should reasonably control it to benefit long-term recovery.

The degree of anxiety was found to be higher in the previous treatment group, which had undergone failed procedures such as unsuccessful MVD and radiofrequency treatment of TN. This group of patients presented with escalated level of anxiety and fear of surgical failure, with their anxiety and depression persisting longer after PBC surgery, negatively impacting their QoL. It has been reported that repeated surgical procedures could induce scar tissue surrounding the trigeminal ganglion region, making these patients more resistant to PBC treatment with reduced treatment efficacy [50, 51]. Over time, we observed that anxiety diminished with positive treatment outcomes, indicating an overall favorable prognosis for this group. These results suggest that pain-related comorbidities should be evaluated and included in the treatment plan. Mental counseling should also be provided when necessary to alleviate patients' concerns, particularly for those with prior interventions. Educating patients about possible surgical outcomes and symptoms such as facial numbness and chewing difficulties following PBC can enhance their adaptability and acceptance of these symptoms.

We conclude that the postoperative outcomes regarding the perception of numbness, acceptance of numbness, improvement of negative emotions, sleep quality, and QoL can vary based on factors such as disease duration, pain perception, preoperative anxiety, and depression levels and previous treatments received by the patient. Hence, it is crucial to consider these factors while deciding on a treatment plan. However, this study has several limitations. First, the impacts of gender, income, and educational levels were not assessed. Postoperative complications, including varying degrees of numbness, herpes labialis, and reduced bite force after PBC—while not severe—are potential contributors to depressive disorders. Evaluating how these factors interact with pain comorbidities requires further investigation. Further investigation is needed to evaluate how these factors interact with pain comorbidities. Additionally, since the study is based on a single-center sample, the findings may be less applicable to a broader population. Larger sample sizes involving multiple centers are necessary to refine the results and provide further insights. Overall, PBC surgery is an effective surgical method, but its efficacy may be compromised if depressive disorders are not considered. Addressing the psychological status of patients and improving QoL after PBC is essential, making these findings vital for individualized comprehensive treatment planning for TN patients.

## Figures and Tables

**Table 1:** Description of TN in the International Classification of Headache Disorders (ICHD).

ICHD-3 trigeminal neuralgia description
A disorder characterized by recurrent unilateral brief electric shock-like pains, abrupt in onset, and termination, limited to the distribution of one or more divisions of the trigeminal nerve and triggered by innocuous stimuli. It may develop without apparent cause or be a result of another diagnosed disorder. Additionally, there may be concomitant continuous pain of moderate intensity within the distribution(s) of the affected nerve division(s).

**Table 2:** Diagnostic criteria of TN in the International Classification of Headache Disorders (ICHD).

ICHD-3 trigeminal neuralgia diagnostic criteria
Recurrent paroxysms of unilateral facial pain in the distribution(s) of one or more divisions of the trigeminal nerve, with no radiation beyond^a^, and fulfilling criteria B and C A. Pain has all of the following characteristics: 1. Lasting from a fraction of a second to 2 min^b^ 2. Severe intensity^c^ 3. Electric shock-like, shooting, stabbing, or sharp in quality B. Precipitated by innocuous stimuli within the affected trigeminal distribution^d^ C. Not better accounted for by another ICHD-3 diagnosis

^a^In a few patients, pain may radiate to another division, but it remains within the trigeminal dermatomes.

^b^Duration can change over time, with paroxysms becoming more prolonged. A minority of patients will report attacks predominantly lasting for >2 min.

^c^Pain may become more severe over time.

^d^Some attacks may be, or appear to be, spontaneous, but there must be a history or finding of pain provoked by innocuous stimuli to meet this criterion. Ideally, the examining clinician should attempt to confirm the history by replicating the triggering phenomenon. However, this may not always be possible because of the patient's refusal, awkward anatomical location of the trigger, and/or other factors.

**Table 3:** Information overview.

	Average ± SD/number (%)
Age (years)	67.74 ± 8.89
Gender	
Male	65 (39.6)
Female	99 (60.4)
Marital status	
Married	159 (97.0)
Widowed	5 (3.0)
Occupation	
Farmar	71 (43.3)
Worker	5 (3.0)
Employe	6 (3.7)
Freelancer	32 (19.5)
Retired	33 (20.1)
Unemployed	17 (10.4)
Affected side	
Left	64 (39.0)
Right	99 (60.4)
Both	1 (0.6)
Presence of other treatments	
Yes	58 (35.4)
No	106 (64.6)
Hypertension	
Yes	79 (48.2)
No	85 (51.8)
Diabetes	
Yes	140 (85.4)
No	24 (14.6)

*Note:* Continuous variables are expressed as mean ± standard deviation, categorical variables are expressed as frequency (%).

**Table 4:** Quartiles and ranges of disease duration and the duration of intraoperative balloon compression.

Items	25%	50%	75%	Range
Duration of intraoperative balloon compression (s)	90	120	150	(72, 240)
Disease duration (Years)	2.50	4.75	8.75	(0.03, 34.00)

**Table 5:** Postoperative scores comparison among duration groups 1 day after surgery.

Items	Duration ≤ 1 year (*N* = 21)	1 < Duration ≤ 5 years (*N* = 73)	Duration > 5 years (*N* = 70)	*F*	*p*
Anxiety	9.14 ± 7.38	9.01 ± 6.55	8.63 ± 5.89	0.087	0.916
Depression	8.86 ± 7.09	8.07 ± 5.82	9.2 ± 6.86	0.562	0.571
Sleep quality	7.52 ± 4.02	8.15 ± 3.77	7.2 ± 3.81	1.123	0.328
Numbness	2.71 ± 0.56^a^	2.42 ± 0.49^b^	2.6 ± 0.62^ab^	2.939	0.056
Numbness acceptance	6.43 ± 0.98^a^	6.99 ± 0.98^b^	6.95 ± 1.04^b^	3.223	0.042

*Note*: An item labelled “a” differs significantly from an item labelled “b.” An item labelled “a” does not differ significantly from an item labelled “ab.” An item labelled “b” does not differ significantly from an item labelled “ab.”

**Table 6:** Postoperative scores comparison among duration groups 1 week after surgery.

Items	Duration ≤ 1 year (*N* = 21)	1 < Duration ≤ 5 years (*N* = 73)	Duration > 5 years (*N* = 70)	*F*	*p*
Anxiety	8.1 ± 5.85	8.4 ± 6.44	7.76 ± 6.2	0.187	0.83
Depression	7.76 ± 6.56	7.48 ± 5.66	7.47 ± 6.23	0.021	0.979
Sleep quality	7.05 ± 4.24	6.67 ± 3.63	5.63 ± 3.85	1.842	0.162
Numbness	2.76 ± 0.7	2.41 ± 0.52	2.54 ± 0.65	2.904	0.058
Numbness acceptance	6.9 ± 1.48	7.04 ± 1.26	7.05 ± 1.25	0.223	0.801
QoL	18.05 ± 1.96	18.53 ± 1.97	18.41 ± 2.04	0.485	0.617

**Table 7:** Postoperative scores comparison among duration groups 1 month after surgery.

Items	Duration ≤ 1 year (*N* = 21)	1 < Duration ≤ 5 years (*N* = 73)	Duration > 5 years (*N* = 70)	*F*	*p*
Anxiety	8.14 ± 6.33	8 ± 7.05	7.17 ± 6.66	0.325	0.723
Depression	7.67 ± 6.46	7.3 ± 6.4	6.83 ± 6.16	0.182	0.833
Sleep quality	5.95 ± 3.56	5.92 ± 3.76	4.97 ± 3.5	1.389	0.252
Numbness	2.57 ± 0.6	2.3 ± 0.52	2.37 ± 0.62	1.817	0.166
Numbness acceptance	7.4 ± 1.48	7.39 ± 1.54	7.29 ± 1.93	0.047	0.954
QoL	19.05 ± 2.52	19.4 ± 2.69	19.3 ± 2.35	0.157	0.854

**Table 8:** Postoperative scores comparison among duration groups 3 months after surgery.

Items	Duration ≤ 1 year (*N* = 21)	1 < Duration ≤ 5 years (*N* = 73)	Duration > 5 years (*N* = 70)	*F*	*p*
Anxiety	8.62 ± 7.61	7.86 ± 7.64	6.56 ± 6.86	0.902	0.408
Depression	7.86 ± 6.02	7.26 ± 7.07	6.47 ± 6.29	0.456	0.635
Sleep quality	5.95 ± 4.19	5.27 ± 4.07	4.49 ± 3.6	1.417	0.245
Numbness	2.52 ± 0.68^a^	2.11 ± 0.43^b^	2.27 ± 0.59^ab^	5.107	0.007
Numbness acceptance	7.64 ± 1.75	7.71 ± 1.7	7.52 ± 2.18	1.222	0.297
QoL	19.48 ± 3.08	19.9 ± 2.77	20.00 ± 2.45	0.311	0.734

*Note*: An item labelled “a” differs significantly from an item labelled “b.” An item labelled “a” does not differ significantly from an item labelled “ab.” An item labelled “b” does not differ significantly from an item labelled “ab.”

**Table 9:** Postoperative scores comparison among duration groups 6 months after surgery.

Items	Duration ≤ 1 year (*N* = 21)	1 < Duration ≤ 5 years (*N* = 73)	Duration > 5 years (*N* = 70)	*F*	*p*
Anxiety	8.48 ± 8.32	7.79 ± 7.9	6.36 ± 7.34	0.915	0.403
Depression	7.81 ± 7.1	7 ± 7.51	6.16 ± 6.51	0.531	0.589
Sleep quality	5.33 ± 4.29	4.81 ± 4.13	4.27 ± 3.74	0.682	0.507
Numbness	2.33 ± 0.73	2.05 ± 0.52	2.11 ± 0.63	1.771	0.173
Numbness acceptance	7.52 ± 2.18	7.92 ± 1.97	8.17 ± 1.72	1.004	0.369
QoL	19.62 ± 3.47	20.4 ± 3.1	20.59 ± 2.53	0.886	0.414

**Table 10:** Postoperative scores comparison among duration groups 12 months after surgery.

Items	Duration ≤ 1 year (*N* = 15)	1 < Duration ≤ 5 years (*N* = 36)	Duration > 5 years (*N* = 42)	*F*	*p*
Anxiety	10 ± 9.1	6.64 ± 8.42	5.62 ± 6.96	1.7	0.188
Depression	8.4 ± 7.89	7.11 ± 8.7	6.07 ± 6.59	0.542	0.583
Sleep quality	5.4 ± 5.03	4.44 ± 4.79	4.07 ± 4.87	0.413	0.663
Numbness	2.4 ± 0.51^a^	2.03 ± 0.61^b^	1.98 ± 0.56^b^	3.144	0.048
Numbness acceptance	7.13 ± 2.33	8.14 ± 2.06	8.29 ± 1.9	1.838	0.165
QoL	19.27 ± 3.81	20.56 ± 3.53	20.88 ± 2.68	1.398	0.252

*Note*: An item labelled “a” differs significantly from an item labelled “b.” An item labelled “a” does not differ significantly from an item labelled “ab.” An item labelled “b” does not differ significantly from an item labelled “ab.”

**Table 11:** Postoperative scores comparison among compression time groups 1 day after surgery.

Items	Time < 90 s (*N* = 42)	90 < Time ≤ 120 s (*N* = 64)	Time > 120 s (*N* = 58)	*F*	*p*
Anxiety	6.52 ± 4.1^a^	10.16 ± 7.43^b^	9.14 ± 6.03^b^	4.398	0.014
Depression	6.76 ± 3.69	9.48 ± 7.29	9.1 ± 6.8	2.538	0.082
Sleep quality	6.4 ± 3.15^a^	8.23 ± 3.98^b^	7.95 ± 3.94^b^	3.237	0.042
Numbness	2.17 ± 0.38^a^	2.59 ± 0.56^b^	2.74 ± 0.58^b^	15.213	<0.001
Numbness acceptance	7.31 ± 0.78^a^	6.75 ± 1.08^b^	6.95 ± 1.04^ab^	3.829	0.024

*Note*: An item labelled “a” differs significantly from an item labelled “b.” An item labelled “a” does not differ significantly from an item labelled “ab.” An item labelled “b” does not differ significantly from an item labelled “ab.”

**Table 12:** Postoperative scores comparison among compression time groups 1 week after surgery.

Items	Time < 90 s (*N* = 42)	90 < Time ≤ 120 s (*N* = 64)	Time > 120 s (*N* = 58)	*F*	*p*
Anxiety	5.9 ± 4.1^a^	8.81 ± 7.15^b^	8.86 ± 6.15^b^	3.565	0.031
Depression	5.76 ± 3.42	8.28 ± 7.04	7.93 ± 6.01	2.511	0.084
Sleep quality	5.07 ± 2.85^a^	6.66 ± 3.93^b^	6.72 ± 4.18^b^	2.866	0.06
Numbness	2.19 ± 0.4^a^	2.61 ± 0.61^b^	2.64 ± 0.67^b^	8.573	<0.001
Numbness acceptance	7.55 ± 0.8^a^	6.91 ± 1.39^b^	7.05 ± 1.25^b^	4.614	0.011
QoL	19.4 ± 1.25^a^	18.02 ± 2.22^b^	18.16 ± 1.95^b^	7.531	0.001

*Note*: An item labelled “a” differs significantly from an item labelled “b.” An item labelled “a” does not differ significantly from an item labelled “ab.” An item labelled “b” does not differ significantly from an item labelled “ab.”

**Table 13:** Postoperative scores comparison among compression time groups 1 month after surgery.

Items	Time < 90 s (*N* = 42)	90 < Time ≤ 120 s (*N* = 64)	Time > 120 s (*N* = 58)	*F*	*p*
Anxiety	5.48 ± 4.15^a^	8.55 ± 7.61^b^	8.28 ± 7.07^b^	3.048	0.05
Depression	5.31 ± 3.35	8.09 ± 7.41	7.43 ± 6.35	2.641	0.074
Sleep	4.26 ± 2.66^a^	5.94 ± 3.62^b^	5.97 ± 4.09^b^	3.474	0.033
Numbness	2.05 ± 0.31^a^	2.48 ± 0.62^b^	2.47 ± 0.6^b^	9.544	<0.001
Numbness acceptance	7.09 ± 1.5^a^	7.29 ± 1.64^b^	8.31 ± 1.14^ab^	4.335	0.015
QoL	20.5 ± 1.47^a^	18.81 ± 2.84^b^	19.00 ± 2.49^b^	6.867	0.001

*Note*: An item labelled “a” differs significantly from an item labelled “b.” An item labelled “a” does not differ significantly from an item labelled “ab.” An item labelled “b” does not differ significantly from an item labelled “ab.”

**Table 14:** Postoperative scores comparison among compression time groups 3 months after surgery.

Items	Time < 90 s (*N* = 42)	90 < Time ≤ 120 s (*N* = 64)	Time > 120 s (*N* = 58)	*F*	*p*
Anxiety	5.05 ± 4.68^a^	8.61 ± 8.22^b^	7.78 ± 7.52^ab^	3.211	0.043
Depression	4.83 ± 3.04^a^	7.98 ± 7.57^b^	7.48 ± 7.04^b^	3.219	0.043
Sleep	3.33 ± 2.51^a^	5.39 ± 3.83^b^	5.84 ± 4.46^b^	5.835	0.004
Numbness	2.02 ± 0.35^a^	2.29 ± 0.58^b^	2.33 ± 0.6^b^	4.346	0.015
Numbness acceptance	7.42 ± 1.87^a^	7.71 ± 1.7^b^	8.74 ± 1.11^b^	3.781	0.025
QoL	20.93 ± 1.83^a^	19.52 ± 2.93^b^	19.55 ± 2.72^b^	4.461	0.013

*Note*: An item labelled “a” differs significantly from an item labelled “b.” An item labelled “a” does not differ significantly from an item labelled “ab.” An item labelled “b” does not differ significantly from an item labelled “ab.”

**Table 15:** Postoperative scores comparison among compression time groups 6 months after surgery.

Items	Time < 90 s (*N* = 42)	90 < Time ≤ 120 s (*N* = 64)	Time > 120 s (*N* = 58)	*F*	*p*
Anxiety	4.67 ± 4.42^a^	8.34 ± 8.22^b^	7.97 ± 8.65^b^	3.341	0.038
Depression	4.38 ± 2.59^a^	7.69 ± 7.91^b^	7.41 ± 7.85^b^	3.308	0.039
Sleep	3.05 ± 2.21^a^	4.97 ± 3.93^b^	5.45 ± 4.69^b^	5.016	0.008
Numbness	1.79 ± 0.42^a^	2.23 ± 0.61^b^	2.22 ± 0.62^b^	9.415	<0.001
Numbness acceptance	8.74 ± 1.11^a^	7.66 ± 2.08^b^	7.78 ± 2.01^b^	4.845	0.009
QoL	21.57 ± 1.68^a^	19.89 ± 3.26^b^	20.05 ± 3.03^b^	4.992	0.008

*Note*: An item labelled “a” differs significantly from an item labelled “b.”

**Table 16:** Postoperative scores comparison among compression time groups 12 months after surgery.

Items	Time < 90 s (*N* = 17)	90 < Time ≤ 120 s (*N* = 34)	Time > 120 s (*N* = 42)	*F*	*p*
Anxiety	2.71 ± 1.61	8.06 ± 8.32	7.26 ± 8.79	2.846	0.063
Depression	3.41 ± 1.54	7.59 ± 7.68	7.64 ± 8.75	2.16	0.121
Sleep quality	1.59 ± 0.8^a^	4.91 ± 4.34^b^	5.19 ± 5.73^b^	3.857	0.025
Numbness	1.76 ± 0.44	2.15 ± 0.61	2.12 ± 0.59	2.853	0.063
Numbness acceptance	9.12 ± 0.6	7.85 ± 2	7.76 ± 2.35	2.992	0.055
QoL	22.18 ± 0.95	19.91 ± 3.47	20.29 ± 3.46	3.067	0.051

*Note*: An item labelled “a” differs significantly from an item labelled “b.”

**Table 17:** Postoperative scores comparison among preoperative pain groups 1 day after surgery.

Items	Pain score ≤ 110 (*N* = 23)	Pain score > 110 (*N* = 141)	*F*	*p*
Anxiety	9.09 ± 6.91	8.83 ± 6.29	0.032	0.858
Depression	7.87 ± 6.69	8.78 ± 6.41	0.395	0.531
Sleep quality	8.74 ± 4.3	7.49 ± 3.73	2.128	0.147
Numbness	2.43 ± 0.51	2.55 ± 0.58	0.855	0.356
Numbness acceptance	6.83 ± 1.15	6.97 ± 1.03	0.383	0.537

**Table 18:** Postoperative scores comparison among preoperative pain groups 1 week after surgery.

Items	Pain score ≤ 110 (*N* = 23)	Pain score > 110 (*N* = 141)	*F*	*p*
Anxiety	8.26 ± 5.89	8.06 ± 6.31	0.021	0.885
Depression	7.04 ± 6.73	7.59 ± 5.88	0.163	0.687
Sleep	6.83 ± 3.64	6.18 ± 3.86	0.556	0.457
Numbness	2.52 ± 0.59	2.51 ± 0.62	0.006	0.936
Numbness acceptance	6.87 ± 1.39	7.09 ± 1.23	0.587	0.445
QoL	22.18 ± 0.95	19.91 ± 3.47	3.067	0.051

**Table 19:** Postoperative scores comparison among preoperative pain groups 1 month after surgery.

Items	Pain score ≤ 110 (*N* = 23)	Pain score > 110 (*N* = 141)	*F*	*p*
Anxiety	9 ± 6.89	7.45 ± 6.75	1.041	0.309
Depression	8.3 ± 7.39	6.96 ± 6.08	0.911	0.341
Sleep	6.48 ± 4.03	5.36 ± 3.56	1.875	0.173
Numbness	2.35 ± 0.49	2.37 ± 0.59	0.026	0.872
Numbness acceptance	7.17 ± 1.85	7.41 ± 1.42	0.505	0.478
QoL	18.61 ± 3.07	19.43 ± 2.4	2.104	0.149

**Table 20:** Postoperative scores comparison among preoperative pain groups 3 months after surgery.

Items	Pain score ≤ 110 (*N* = 23)	Pain score > 110 (*N* = 141)	*F*	*p*
Anxiety	9.48 ± 8.35	7.06 ± 7.1	2.172	0.142
Depression	7.78 ± 6.81	6.87 ± 6.58	0.375	0.541
Sleep	6.78 ± 4.7	4.74 ± 3.7	5.584	0.019
Numbness	2.26 ± 0.45	2.23 ± 0.57	0.068	0.795
Numbness acceptance	7.04 ± 2.38	7.82 ± 1.54	4.251	0.041
QoL	19.13 ± 3.32	20.01 ± 2.54	2.186	0.141

**Table 21:** Postoperative scores comparison among preoperative pain groups 6 months after surgery.

Items	Pain score ≤ 110 (*N* = 23)	Pain score > 110 (*N* = 141)	*F*	*p*
Anxiety	9.78 ± 9.38	6.86 ± 7.37	2.875	0.092
Depression	7.96 ± 8.46	6.55 ± 6.78	0.796	0.374
Sleep	6.26 ± 4.9	4.38 ± 3.76	4.501	0.035
Numbness	2.22 ± 0.42	2.1 ± 0.63	0.763	0.384
Numbness acceptance	6.96 ± 2.72	8.14 ± 1.68	8.064	0.005
QoL	19.39 ± 3.89	20.54 ± 2.71	3.092	0.081

**Table 22:** Postoperative scores comparison among preoperative pain groups 12 months after surgery.

Items	Pain score ≤ 110 (*N* = 13)	Pain score > 110 (*N* = 80)	*F*	*p*
Pain	0 ± 0	0.8 ± 6.27	0.209	0.648
Anxiety	10.85 ± 11.78	6.05 ± 7.04	4.195	0.043
Depression	10.69 ± 11	6.23 ± 6.84	3.946	0.05
Sleep	6.77 ± 5.69	4.05 ± 4.61	3.645	0.059
Numbness	2.31 ± 0.63	2.03 ± 0.57	2.647	0.107
Numbness acceptance	6.85 ± 3.29	8.24 ± 1.73	5.38	0.023
QoL	19.08 ± 4.41	20.73 ± 2.98	2.963	0.089

**Table 23:** Postoperative scores comparison among preoperative anxiety groups 1 day after surgery.

Items	Anxiety score ≤ 8 (*N* = 72)	8 < Anxiety score ≤ 20 (*N* = 73)	Anxiety score > 20 (*N* = 19)	*F*	*p*
Anxiety	4.38 ± 1.67^a^	10.6 ± 3.76^b^	19.21 ± 9.62^c^	103.521	<0.001
Depression	4.96 ± 2.78^a^	9.9 ± 5.25^b^	17.84 ± 9.16^c^	53.752	<0.001
Sleep	5.76 ± 2.83^a^	8.49 ± 3.56^b^	11.68 ± 3.99^c^	28.145	<0.001
Numbness	2.44 ± 0.53	2.62 ± 0.59	2.58 ± 0.61	1.731	0.18
Numbness acceptance	7.42 ± 0.73^a^	6.7 ± 1.06^b^	6.95 ± 1.04^b^	17.835	<0.001

*Note*: Items with different letters (e.g. “a” and “b”, “a” and “c”) have significant difference. Items sharing the same letter (e.g. “a” and “a”, “a” and “ab”) have no significant difference.

**Table 24:** Postoperative scores comparison among preoperative anxiety groups 1 week after surgery.

Items	Anxiety score ≤ 8 (*N* = 72)	8 < Anxiety score ≤ 20 (*N* = 73)	Anxiety score > 20 (*N* = 19)	*F*	*p*
Anxiety	4.15 ± 1.98^a^	9.81 ± 4.29^b^	16.37 ± 10.78^ab^	57.316	<0.001
Depression	4.47 ± 2.1^a^	8.59 ± 4.91^b^	14.89 ± 10.57^c^	35.444	<0.001
Sleep	4.51 ± 2.39^a^	7.23 ± 3.93^b^	9.26 ± 4.74^b^	19.277	<0.001
Numbness	2.4 ± 0.55	2.62 ± 0.64	2.53 ± 0.7	2.252	0.109
Numbness acceptance	7.61 ± 0.83^a^	6.11 ± 1.37^b^	7.05 ± 1.25^b^	17.78	<0.001
QoL	19.26 ± 1.41^a^	18.07 ± 1.87^b^	16.58 ± 2.65^b^	19.256	<0.001

*Note*: Items with different letters (e.g. “a” and “b”, “a” and “c”) have significant difference. Items sharing the same letter (e.g. “a” and “a”, “a” and “ab”) have no significant difference.

**Table 25:** Postoperative scores comparison among preoperative anxiety groups 1 month after surgery.

Items	Anxiety score ≤ 8 (*N* = 72)	8 < Anxiety score ≤ 20 (*N* = 73)	Anxiety score > 20 (*N* = 19)	*F*	*p*
Anxiety	3.68 ± 2.21^a^	9.45 ± 5.32^b^	15.89 ± 11.61^b^	44.573	<0.001
Depression	4.21 ± 2.19^a^	8.4 ± 5.51^b^	13.47 ± 11.53^b^	24.483	<0.001
Sleep	3.94 ± 2.3^a^	6.47 ± 3.87^b^	7.84 ± 4.45^b^	15.423	<0.001
Numbness	2.25 ± 0.5	2.48 ± 0.63	2.37 ± 0.6	2.947	0.055
Numbness acceptance	6.93 ± 1.68^a^	6.63 ± 1.67^b^	8.46 ± 0.9^b^	14.754	<0.001
QoL	20.31 ± 1.58^a^	18.89 ± 2.52^b^	17.16 ± 3.52^b^	16.178	<0.001

*Note*: An item labelled “a” differs significantly from an item labelled “b.”

**Table 26:** Postoperative scores comparison among preoperative anxiety groups 3 month after surgery.

Items	Anxiety score ≤ 8 (*N* = 72)	8 < Anxiety score ≤ 20 (*N* = 73)	Anxiety score > 20 (*N* = 19)	*F*	*p*
Anxiety	3.25 ± 2.62^a^	9.16 ± 6.12^b^	16.37 ± 11.88^b^	42.172	<0.001
Depression	3.9 ± 2.08^a^	8.52 ± 6.43^b^	12.89 ± 11.27^b^	21.954	<0.001
Sleep	3.43 ± 2.66^a^	5.9 ± 4.14^b^	7.68 ± 4.52^b^	14.275	<0.001
Numbness	2.08 ± 0.4^a^	2.36 ± 0.66^b^	2.32 ± 0.48^ab^	5.063	0.007
Numbness acceptance	7.29 ± 1.87^a^	7.71 ± 1.7^b^	8.86 ± 0.92^b^	16.523	<0.001
QoL	20.97 ± 1.43^a^	19.37 ± 2.88^b^	17.79 ± 3.57^b^	15.561	<0.001

*Note*: An item labelled “a” differs significantly from an item labelled “b.” An item labelled “a” does not differ significantly from an item labelled “ab.” An item labelled “b” does not differ significantly from an item labelled “ab.”

**Table 27:** Postoperative scores comparison among preoperative anxiety groups 6 month after surgery.

Items	Anxiety score ≤ 8 (*N* = 72)	8 < Anxiety score ≤ 20 (*N* = 73)	Anxiety score > 20 (*N* = 19)	*F*	*p*
Anxiety	3.25 ± 3.03^a^	8.97 ± 7.04^b^	15.95 ± 12.19^b^	32.77	<0.001
Depression	3.64 ± 2.06^a^	8.12 ± 7.11^b^	13.21 ± 11.72^b^	20.416	<0.001
Sleep	3.04 ± 2.54^a^	5.53 ± 4.33^b^	7.32 ± 4.67^b^	13.837	<0.001
Numbness	1.93 ± 0.45^a^	2.27 ± 0.67^b^	2.21 ± 0.63^ab^	6.619	0.002
Numbness acceptance	8.86 ± 0.92^a^	7.44 ± 2.05^b^	6.68 ± 2.52^b^	18.443	<0.001
QoL	21.65 ± 1.38^a^	19.74 ± 3.13^b^	18 ± 4.06^b^	18.006	<0.001

*Note*: An item labelled “a” differs significantly from an item labelled “b.” An item labelled “a” does not differ significantly from an item labelled “ab.” An item labelled “b” does not differ significantly from an item labelled “ab.”

**Table 28:** Postoperative scores comparison among preoperative anxiety groups 12 month after surgery.

Items	Anxiety score ≤ 8 (*N* = 41)	8 < Anxiety score ≤ 20 (*N* = 39)	Anxiety score > 20 (*N* = 13)	*F*	*p*
Anxiety	2.39 ± 0.67^a^	8.64 ± 7.99^b^	14.62 ± 11.81^b^	18.847	<0.001
Depression	2.78 ± 1.26^a^	9.46 ± 8.73^b^	11.85 ± 9.75^b^	13.932	<0.001
Sleep	2.37 ± 2.53^a^	5.56 ± 5.7^b^	7.54 ± 5.06^b^	8.779	<0.001
Numbness	1.88 ± 0.46^a^	2.21 ± 0.66^b^	2.23 ± 0.6^ab^	3.958	0.023
Numbness acceptance	9.17 ± 0.5^a^	7.23 ± 2.32^b^	6.92 ± 2.57^b^	14.432	<0.001
QoL	22.37 ± 0.66^a^	19.33 ± 3.53^b^	18.08 ± 4.07^b^	17.72	<0.001

*Note*: An item labelled “a” differs significantly from an item labelled “b.” An item labelled “a” does not differ significantly from an item labelled “ab.” An item labelled “b” does not differ significantly from an item labelled “ab.”

**Table 29:** Postoperative scores comparison among preoperative depression groups 1 day after surgery.

Items	Depression score ≤ 8 (*N* = 80)	8 < Depression score ≤ 20 (*N* = 66)	Depression score > 20 (*N* = 18)	*F*	*p*
Anxiety	5.14 ± 3.01^a^	11.33 ± 5.03^b^	16.39 ± 9.98^b^	50.285	<0.001
Depression	4.46 ± 1.79^a^	10.88 ± 3.58^b^	19.11 ± 10.74^c^	97.775	<0.001
Sleep	5.76 ± 2.8^a^	8.95 ± 3.64^b^	11.39 ± 3.88^b^	30.132	<0.001
Numbness	2.44 ± 0.52	2.62 ± 0.6	2.67 ± 0.59	2.456	0.089
Numbness acceptance	7.38 ± 0.86^a^	6.73 ± 0.89^b^	6.95 ± 1.04^c^	21.905	<0.001

*Note*: Items with different letters (e.g. “a” and “b”, “a” and “c”) have significant difference. Items sharing the same letter (e.g. “a” and “a”, “a” and “ab”) have no significant difference.

**Table 30:** Postoperative scores comparison among preoperative depression groups 1 week after surgery.

Items	Depression score ≤ 8 (*N* = 80)	8 < Depression score ≤ 20 (*N* = 66)	Depression score > 20 (*N* = 18)	*F*	*p*
Anxiety	5.11 ± 3.72^a^	9.74 ± 5.23^b^	15.22 ± 9.88^b^	32.093	<0.001
Depression	4.39 ± 2.2^a^	8.55 ± 3.91^b^	17.61 ± 10.23^b^	68.598	<0.001
Sleep	4.74 ± 2.77^a^	7.12 ± 3.68^b^	10 ± 4.9^c^	20.656	<0.001
Numbness	2.4 ± 0.54	2.59 ± 0.66	2.72 ± 0.67	3.027	0.051
Numbness acceptance	7.49 ± 1.09^a^	5.94 ± 1.47^b^	7.05 ± 1.25^c^	15.193	<0.001
QoL	19.19 ± 1.48^a^	18.03 ± 1.93^b^	16.44 ± 2.48^b^	19.777	<0.001

*Note*: Items with different letters (e.g. “a” and “b”, “a” and “c”) have significant difference. Items sharing the same letter (e.g. “a” and “a”, “a” and “ab”) have no significant difference.

**Table 31:** Postoperative scores comparison among preoperative depression groups 1 month after surgery.

Items	Depression score ≤ 8 (*N* = 80)	8 < Depression score ≤ 20 (*N* = 66)	Depression score > 20 (*N* = 18)	*F*	*p*
Anxiety	4.59 ± 3.94^a^	9.11 ± 5.82^b^	16.06 ± 10.53^c^	32.772	<0.001
Depression	4.4 ± 3.19^a^	7.88 ± 4.3^b^	16.67 ± 11.23^c^	44.078	<0.001
Sleep	3.91 ± 2.37^a^	6.44 ± 3.54^b^	9.28 ± 4.73^b^	25.389	<0.001
Numbness	2.28 ± 0.5	2.41 ± 0.61	2.61 ± 0.7	2.879	0.059
Numbness acceptance	7.14 ± 1.42^a^	6.17 ± 1.92^b^	8.29 ± 1.29^b^	12.468	<0.001
QoL	20.11 ± 1.9^a^	19 ± 2.39^b^	16.89 ± 3.53^b^	15.191	<0.001

*Note*: Items with different letters (e.g. “a” and “b”, “a” and “c”) have significant difference. Items sharing the same letter (e.g. “a” and “a”, “a” and “ab”) have no significant difference.

**Table 32:** Postoperative scores comparison among preoperative depression groups 3 months after surgery.

Items	Depression score ≤ 8 (*N* = 80)	8 < Depression score ≤ 20 (*N* = 66)	Depression score > 20 (*N* = 18)	*F*	*p*
Anxiety	4.14 ± 4.33^a^	8.94 ± 6.64^b^	16.28 ± 10.68^c^	31.071	<0.001
Depression	4.13 ± 2.95^a^	7.76 ± 5.38^b^	17 ± 10.92^c^	43.803	<0.001
Sleep	3.45 ± 2.77^a^	5.65 ± 3.53^b^	9.72 ± 5.11^c^	26.893	<0.001
Numbness	2.11 ± 0.42^a^	2.32 ± 0.66^ab^	2.47 ± 0.51^b^	4.469	0.013
Numbness acceptance	7.44 ± 1.72^a^	7.71 ± 1.7^b^	8.57 ± 1.43^b^	15.154	<0.001
QoL	20.76 ± 1.88^a^	19.53 ± 2.73^b^	17.33 ± 3.5^b^	15.48	<0.001

*Note*: Items with different letters (e.g. “a” and “b”, “a” and “c”) have significant difference. Items sharing the same letter (e.g. “a” and “a”, “a” and “ab”) have no significant difference.

**Table 33:** Postoperative scores comparison among preoperative depression groups 6 months after surgery.

Items	Depression score ≤ 8 (*N* = 80)	8 < Depression score ≤ 20 (*N* = 66)	Depression score > 20 (*N* = 18)	*F*	*p*
Anxiety	4.1 ± 4.66^a^	8.61 ± 7.53^b^	16.44 ± 10.46^c^	27.014	<0.001
Depression	3.81 ± 3.02^a^	7.44 ± 6.26^b^	17.22 ± 11.07^c^	40.565	<0.001
Sleep	3.18 ± 2.85^a^	5.36 ± 4.1^b^	8.56 ± 4.63^c^	18.509	<0.001
Numbness	1.98 ± 0.45^a^	2.2 ± 0.66^a^	2.44 ± 0.78^a^	5.821	0.004
Numbness acceptance	8.57 ± 1.43^a^	7.68 ± 1.93^b^	6.39 ± 2.45^b^	12.685	<0.001
QoL	21.41 ± 1.92^a^	20.05 ± 2.86^b^	17 ± 3.99^c^	21.986	<0.001

*Note*: Items with different letters (e.g. “a” and “b”, “a” and “c”) have significant difference. Items sharing the same letter (e.g. “a” and “a”, “a” and “ab”) have no significant difference.

**Table 34:** Postoperative scores comparison among preoperative depression groups 12 months after surgery.

Items	Depression score ≤ 8 (*N* = 45)	8 < Depression score ≤ 20 (*N* = 38)	Depression score > 20 (*N* = 10)	*F*	*p*
Anxiety	3.13 ± 3.82^a^	8.53 ± 8.67^b^	16 ± 9.71^b^	27.014	<0.001
Depression	3.18 ± 3^a^	8.47 ± 8.03^b^	17.2 ± 9.5^b^	40.565	<0.001
Sleep	2.58 ± 2.94^a^	5.58 ± 5.52^b^	8.4 ± 5.54^b^	18.509	<0.001
Numbness	1.93 ± 0.45^a^	2.03 ± 0.59^a^	2.8 ± 0.63^b^	5.821	<0.001
Numbness acceptance	8.96 ± 1.04^a^	7.5 ± 2.37^b^	6 ± 2.16^b^	13.681	<0.001
QoL	22.13 ± 1.39^a^	19.74 ± 3.37^b^	16 ± 3.56^c^	21.986	<0.001

*Note*: Items with different letters (e.g. “a” and “b”, “a” and “c”) have significant difference. Items sharing the same letter (e.g. “a” and “a”, “a” and “ab”) have no significant difference.

**Table 35:** Postoperative scores comparison among other previous treatment groups 1 day after surgery.

Items	No (*N* = 58)	Yes (*N* = 106)	*F*	*p*
Anxiety	7.88 ± 5.35	10.67 ± 7.59	7.538	0.007
Depression	8.03 ± 5.33	9.79 ± 8	2.853	0.093
Sleep	7.52 ± 3.84	7.93 ± 3.81	0.434	0.511
Numbness	2.46 ± 0.59	2.67 ± 0.51	5.246	0.023
Numbness acceptance	7.04 ± 0.96	6.79 ± 1.18	2.072	0.152

**Table 36:** Postoperative scores comparison among other previous treatment groups 1 week after surgery.

Items	No (*N* = 58)	Yes (*N* = 106)	*F*	*p*
Anxiety	6.91 ± 4.59	10.24 ± 8.06	11.423	0.001
Depression	6.49 ± 4.15	9.38 ± 8.08	9.167	0.003
Sleep	6.08 ± 3.55	6.64 ± 4.28	0.811	0.369
Numbness	2.42 ± 0.65	2.67 ± 0.51	6.36	0.013
Numbness acceptance	7.19 ± 1.18	6.81 ± 1.34	3.489	0.064
QoL	18.7 ± 1.81	17.91 ± 2.22	5.997	0.015

**Table 37:** Postoperative scores comparison among other previous treatment groups 1 month after surgery.

Items	No (*N* = 58)	Yes (*N* = 106)	*F*	*p*
Anxiety	6.62 ± 4.99	9.57 ± 8.92	7.376	0.007
Depression	6.24 ± 4.57	8.81 ± 8.35	6.526	0.012
Sleep	5.38 ± 3.32	5.78 ± 4.17	0.449	0.504
Numbness	2.31 ± 0.61	2.47 ± 0.5	2.716	0.101
Numbness acceptance	7.48 ± 1.36	7.19 ± 1.68	1.452	0.23
QoL	19.63 ± 2.22	18.72 ± 2.91	5.015	0.026

**Table 38:** Postoperative scores comparison among other previous treatment groups 3 months after surgery.

Items	No (*N* = 58)	Yes (*N* = 106)	*F*	*p*
Anxiety	6.21 ± 5.73	9.59 ± 9.22	8.367	0.004
Depression	6.08 ± 4.8	8.67 ± 8.81	5.944	0.016
Sleep	4.87 ± 3.74	5.31 ± 4.2	0.48	0.489
Numbness	2.18 ± 0.57	2.33 ± 0.51	2.676	0.104
Numbness acceptance	7.89 ± 1.55	7.4 ± 1.91	3.169	0.077
QoL	20.2 ± 2.42	19.33 ± 3.01	4.067	0.045

**Table 39:** Postoperative scores comparison among other previous treatment groups 6 months after surgery.

Items	No (*N* = 58)	Yes (*N* = 106)	*F*	*p*
Anxiety	6.19 ± 6.27	9.24 ± 9.57	6.054	0.015
Depression	5.86 ± 5.39	8.36 ± 9.14	4.874	0.029
Sleep	4.42 ± 3.8	5.07 ± 4.28	1.013	0.316
Numbness	2.06 ± 0.66	2.22 ± 0.46	2.952	0.088
Numbness acceptance	8.2 ± 1.75	7.57 ± 2.1	4.209	0.042
QoL	20.71 ± 2.69	19.78 ± 3.23	3.882	0.051

**Table 40:** Postoperative scores comparison among other previous treatment groups 12 months after surgery.

Items	No (*N* = 35)	Yes (*N* = 58)	*F*	*p*
Anxiety	6.12 ± 7.28	7.71 ± 9.01	0.873	0.353
Depression	6.09 ± 6.98	8.11 ± 8.58	1.547	0.217
Sleep	4.21 ± 4.31	4.8 ± 5.64	0.327	0.569
Numbness	2.03 ± 0.65	2.11 ± 0.47	0.402	0.528
Numbness acceptance	8.21 ± 1.9	7.77 ± 2.29	0.982	0.324
QoL	20.69 ± 3.1	20.17 ± 3.48	0.557	0.457

## Data Availability

All statistical results are included in the article. Further inquiry about the data can be made upon requesting the corresponding author Chunran Zhu at zhuchunran2020@163.com.

## Nomenclature

AICA: Anterior inferior cerebellar artery
HAMA: Hamilton Anxiety Scale
HAMD: Hamilton Depression Scale
LSI-B: Life Satisfaction Index-B
MVD: Microvascular decompression
PBC: Percutaneous balloon compression
PICA: Posterior inferior cerebellar artery
PSQI: Pittsburgh Sleep Quality Index
QoL: Quality of life
SD: Standard deviation
SCA: Superior cerebellar artery
TN: Trigeminal neuralgia.
